# Changes of plasma cytokines and chemokines expression level in nasopharyngeal carcinoma patients after treatment with definitive intensity-modulated radiotherapy (IMRT)

**DOI:** 10.1371/journal.pone.0172264

**Published:** 2017-02-16

**Authors:** Ya-bin Jin, Guo-yi Zhang, Kai-Rong Lin, Xiang-ping Chen, Jin-Huan Cui, Yue-jian Wang, Wei Luo

**Affiliations:** 1 Clinical Research Institute, Foshan Hospital, Sun Yat-sen University, Foshan, Guangdong, China; 2 Head and Neck Cancer Research, Department of Otolaryngology-Head and Neck Surgery, Foshan Hospital, Sun Yat-sen University, Foshan, Guangdong, China; 3 Cancer Center, Foshan Hospital, Sun Yat-sen University, Foshan, Guangdong, China; 4 Otolaryngology head and neck surgery, Foshan Hospital, Sun Yat-sen University, Foshan, Guangdong, China; Taipei Medical University, TAIWAN

## Abstract

**Background:**

Potential clinical application values of certain cytokines and chemokines that participate in the process of tumor growth, invasion, and metastasis have been reported. However, there still lack of biomarkers for a great many of malignancy. This study identified cytokines or chemokines involved in the occurrence and development of nasopharyngeal carcinoma (NPC), which might be a biomarker for noninvasive early diagnosis.

**Methods:**

The plasma levels of 19 cytokines and chemokines were detected by the luminex liquid array-based multiplexed immunoassays in 39 NPC patients before and after treatment by definitive intensity-modulated radiotherapy (IMRT).

**Results:**

Plasma levels of almost all of the 19 cytokines and chemokines in NPC patients were higher than healthy controls, while only IFN-γ, IL-1b IL-6, MCP-1, TNF-α, FKN, IL-12P70, IL-2, IL-5 and IP-10 showed significant differences. However, expression levels of most of the 19 cytokines and chemokines decreased after therapy, especially IFN-γ, IL-10, IL-1b, IL-6, IL-8, MCP-1, TNF-α, VEGF, IL-17A, IL-2, IL-5 and MIP-1b, have a dramatic decline. Taking together, plasma levels of IFN-γ, IL-1b, IL-6, MCP-1, TNF-α, IL-2 and IL-5 are significantly increased in NPC patients and dramatically decreased after treatment, suggesting these cytokines and chemokines might play important roles in the progress of NPC. More interestingly, the expression level of MPC-1 is significantly associated with clinical stage.

**Conclusion:**

MCP-1 might involve in the genesis and development process of NPC, which might serve as a noninvasive biomarker for early diagnosis.

## Introduction

Recent researches have revealed that not only tumor cells themselves, but also many host factors such as immune status in tumor microenvironment have deep influence on formation, development and biological behavior of tumor [1]. Many cytokines/chemokines and their receptors have been found in cancers, which are networked and involved in regulating the immune response of tumor microenvironment to promote tumor cell growth, survival, invasion and transfer [2].

Nasopharyngeal carcinoma (NPC) is a squamous cell carcinoma arising from the epithelial lining of the nasopharynx, which has a high incidence in Southern China and been called “Guangdong Tumor” because of its unique geographic distribution characteristic [3]. The vast majority of NPC patients are diagnosed as advanced metastasis disease due to its hidden pathogenic site [4]. Blood biomarkers may contribute to noninvasive early diagnosis of NPC.

Since numerous immune cells and their secreting cytokines and chemokines with regulating functions have been found in nasopharyngeal carcinoma, some cytokines/chemokines showed the potential of served as biomarkers for diagnosis and treatment of NPC [5]. High serum expression levels of CCL2 (chemokine C–C motif ligand 2 or monocyte chemotactic protein-1, MCP-1) and TNF-α have been reported can predicted bone invasion, distant metastasis and poor overall survival in NPC patients [6]. Plasma Epstein-Barr (EB) viral load and level of interleukin (IL)-8 and IL-10 had been found associated with the stages of NPC and consider as prognostic indicators for NPC[7]. However, there still lack of biomarker for noninvasive early diagnosis of NPC.

In the present study, expression levels of 19 plasma cytokines and chemokines have been detected by luminex liquid array-based multiplexed immunoassays in 39 NPC patients before and after treatment by definitive intensity-modulated radiotherapy (IMRT). Results show that the expression levels of almost all of 19 cytokines and chemokines are higher than healthy controls, and 10 of them increase significantly. However, most of these cytokines and chemokines decrease after therapy, and 12 of them have a dramatic decline. Taking together, plasma levels of 7 cytokines and chemokines are significantly increase in NPC patients and dramatic decrease after treatment, suggesting they might play important roles in the progress of NPC. More interestingly, one of them showed significantly associated with clinical stage, and might serve as a noninvasive biomarker for NPC early diagnosis.

## Materials and methods

### Study population

A total of 39 ethnic Chinese patients from Foshan Hospital of *Sun Yat-sen University*, were initially enrolled in this study from Dec.25.2012 to May.13.2015. All were newly-diagnosed with non-metastatic NPC patients and subsequently treated with definitive intensity-modulated radiotherapy (IMRT), which is the preferred choice of treatment for NPC patients with a favorable toxicity profile and achieves very high loco regional control [8]. All patients underwent pretreatment evaluations, including MRI of the neck and nasopharynx, chest radiography, abdominal sonography and whole-body bone scan, and obtained good therapeutic efficacy after treatment with IMRT. Medical records and imaging results were reviewed, and all patients were restaged according to the AJCC tumor-node-metastases (TNM) staging system, 2010. Besides, sixty-four healthy individuals were included as controls, which showed no significant difference on sexuality, age, smoking status, and history of alcohol intake comparing with NPC patients (Chi-square test).

### Ethics statement

This study was conducted in accordance with the Declaration of Helsinki. All participants agreed and signed informed consent forms prior to participation. The study was approved by the Ethics Committee at Sun Yat-Sen University.

### Plasma samples

Two milliliter peripheral blood were respectively collected from NPC patients before and after treatment and healthy controls in the presence of EDTA anti-coagulant and centrifuged for 10 minutes at 1000×*g*. Plasma samples were separated and kept at -80℃ until use. Prior to cytokine assays, frozen plasma samples were thawed completely, mixed well by vortexing, and centrifuged for 10 minutes at 10000×*g* to remove particulates.

### Cell culture supernatant

PBMC was isolated from 2 ml of peripheral blood by Ficoll-Paque, and then 5×10^5^/200ul of cells were added in each well of 96 wells plate and cultured by RPMI-1640 with 10% fetal bovine serum, after incubated at 37℃, 5% CO_2_ for 48h, the cell supernatants were collected and stored at -80℃ until use.

### Multiplexed cytokine assay

The milliplex map kit containing magnetic beads for quantification of 19 human cytokines and chemokines was purchased from Merck Millipore (Darmstadt, Germany). Multicytokine analyses were performed using Luminex technology. Sensitivity and specificity of the assay were calculated. Assays were performed according to the manufacturer’s instructions. Briefly, after plates were pre-wet, 50 μl of precombined beads was added and washed twice. Plasma samples (25 μl) were diluted 1:1 with serum matrix and added to the plate. The plate was shaken for 30 seconds at 500 rpm and then incubated for one hour on a plate shaker at 700 rpm at room temperature. Plates were washed twice, 25μl of detection antibody was added per well, and plates were incubated for one hour on a plate shaker. 50μl of strepatavidin-PE conjugate was added to each well, and the plate was shaken at 500 rpm for 30 minutes at room temperature. Finally, plates were washed three times and 150 μl of sheath fluid were added to each well. Plates were read using a Luminex machine (Luminex, Austin, USA). Data was analyzed according to the Luminex Instrument instructions. ProcartaPlex Analyst 1.0 was used. A standard curve for each cytokine was generated by mixing known concentrations of recombinant human cytokines.

### Statistical

GraphPad Prism 5 was used to generate plots and SPSS 17.0 was used to perform statistical analyses. Shapiro-Wilk was chosen to conducte the normality test of our data. A paired t-test was performed to compare the cytokines/chemokines between pre- and post-treatment when our data was accorded with normal distribution, otherwise Wilcoxon Signed Rank test was used. The Mann-Whitney rank test was used to compare samples between pre-treatment patients group and healthy control group. Chi-square test was used to compare categorical data between groups. One-way analysis of variance (ANOVA) was used to compare cytokines/chemokines of the NPC patients with different clinical stages. Limited Slip Differential (LSD) post-hoc tests were performed between every two stages. P values < 0.05 were considered statistically significant.

## Results

### Description of demographic characteristics

Thirty-nine subjects diagnosed as NPC without chronic diseases (such as heart failure, autoimmune diseases, and other bacterial or viral infections) were enrolled in the prospective study. NPC patients were classified according to the UICC (2002) staging system of NPC, of whom 2 with stage I, 8 with stage II, 12 with stage III and 17 with stage IV. In addition, the distributions of NPC patients based on other classification systems are also given in Table 1.

**Table 1:** Clinical characteristics of NPC patients.

Characteristics	NPC patients (n = 39)
**Age**	
≥40	18
<40	21
**Gender**	
Male	33
Female	6
**Smoking status**	
No	23
Yes	16
**History of alcohol intake**	
No	24
Yes	15
**Clinical staging**	
I	2
II	8
III	12
IV_a+b_	17
**T staging**	
T1	5
T2	12
T3	10
T4	12
**N staging**	
N0	8
N1	9
N2	16
N3	6
**M staging**	
M0	39
M1	0
**Co-morbidities**	
Yes	15
No	24
**Histological grade**	
non-keratinizing carcinoma	39
keratinizing carcinoma	0

### Plasma cytokine/chemokine expression levels in NPC patients and healthy controls

**Fig 1:**
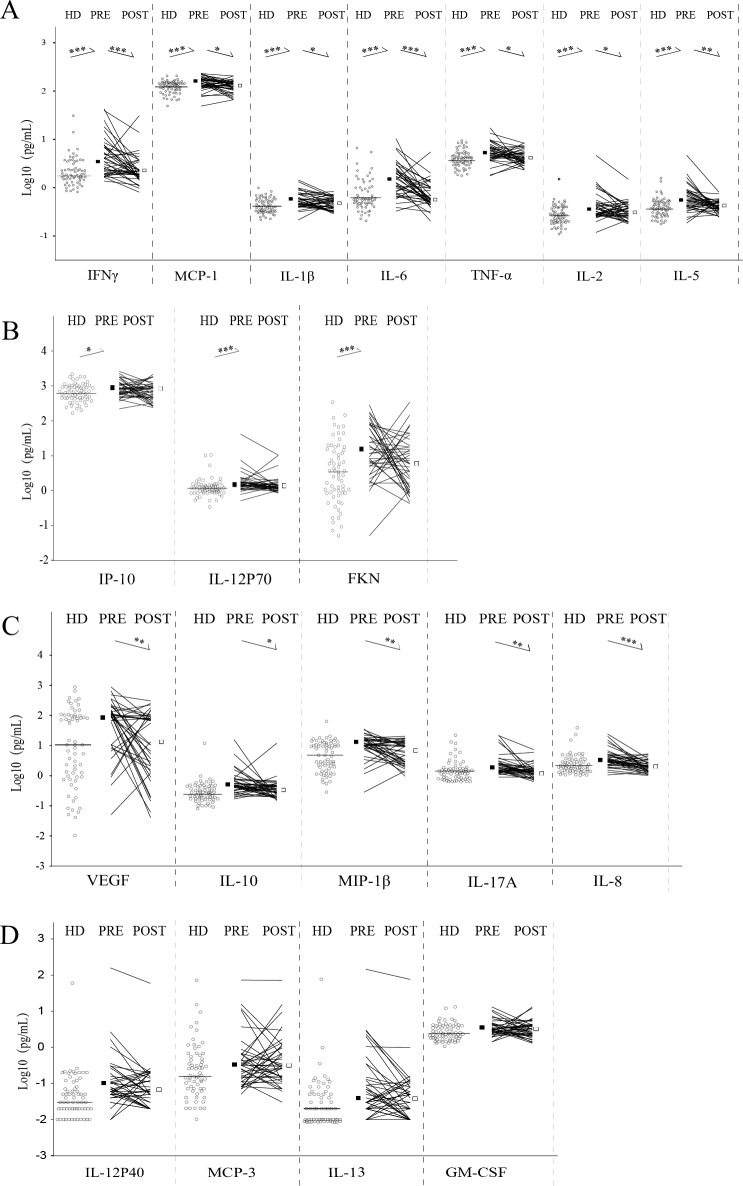
Expression levels of 19 plasma cytokines and chemokines in NPC patients before and after therapy. A. The ones increasing in NPC patients and declining after therapy. B. The ones increasing in NPC patients but no change after therapy. C. The ones no change in NPC patients but decreasing after therapy. D. The ones no change before and after therapy. Total 39 NPC patients (PRE: pre-treated samples; POST: post-treated samples) and 64 healthy donor (HD). The plasma cytokine/chemokine contents (pg/ml) had been transformed by log 10 to correct the non-normal data. The horizontal line (HD) and squares (PRE and POST) represent the median in each group. *P<0.05; **P<0.01; ***P<0.001.

Luminex was used to determine the expression level of plasma cytokines and chemokines in NPC patients and healthy samples. Plasma levels of cytokines and chemokines between NPC patients and healthy donors were compared, and the results showed that the expression levels of most cytokines and chemokines are increase in NPC patients but only 10 of 19 have statistical significances, namely IFN-γ (*P*<0.001), IL-1b (*P* = 0.01), IL-6 (*P*<0.001), MCP-1 (*P*<0.001), TNF-α (*P*<0.001), FKN (*P*<0.001), IL-12P70 (*P*<0.001), IL-2 (*P* = 0.001), IL-5 (*P*<0.001), IP-10 (*P* = 0.04) (Fig 1A and 1B).

### Plasma cytokine/chemokine expression levels in NPC patients before and after treatment

The change of plasma levels of cytokines and chemokines after treatment has been evaluated. Almost all of the 19 cytokines and chemokines decrease after treatment, and 12 of them decline dramatically, which has been shown in Fig 1A and 1C. Taken together, IFN-γ, IL-1b, IL-6, MCP-1, TNF-α, IL-2 and IL-5 are significantly higher in pre-treated NPC patients and decrease after therapy (Fig 1A), which may play important roles in the development of NPC.

### Relationship between plasma levels of cytokine/chemokine and clinical stages

**Fig 2:**
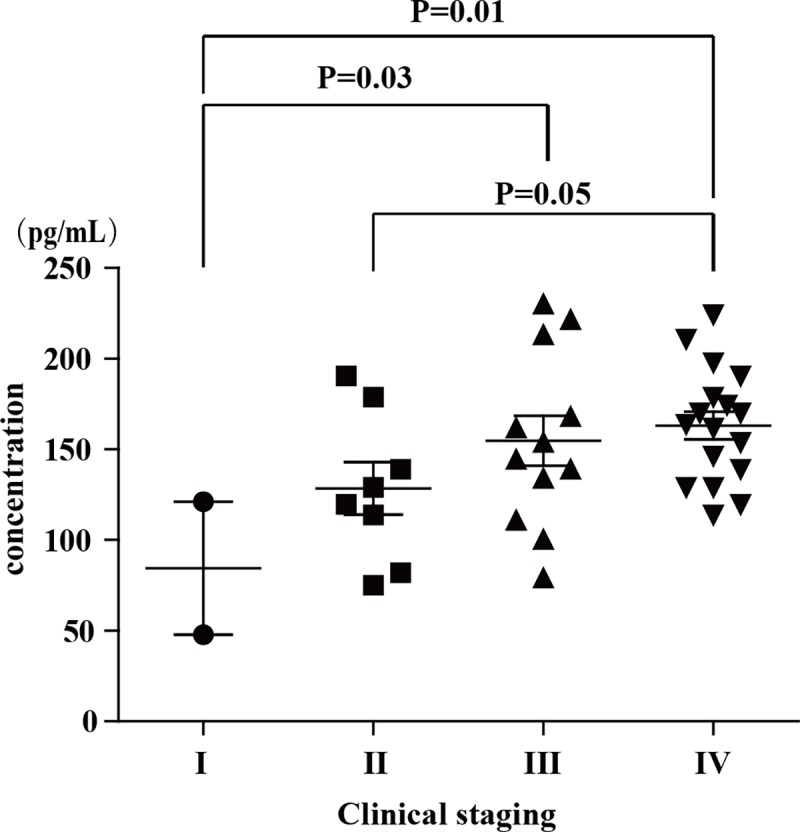
Expression levels of MCP-1 in NPC patients with different stages. Data was analyzed by one-way analysis of variance (ANOVA). Limited Slip Differential (LSD) post-hoc tests were performed between every two stages. The error bars indicate the mean with the SEM.

The relationship between plasma levels of cytokines and chemokines and clinical stages has been analyzed. The expression level of MPC-1 is significantly associated with clinical stage, there is significant difference between four clinical stages (P = 0.034, one-way ANOVA, Fig 2), suggesting that MCP-1 might be a biomarker in the genesis and development process of NPC.

## Discussion

NPC is a multifactorial malignancy disease, and immune state may be a crucial factor in genesis and development of NPC [5]. Cytokines and chemokines secreted by immune cells play important roles in immune response by mutual influence and interaction with each other [9]. Many previous literatures had revealed that several cytokines and chemokines were associated with the development of NPC and played indispensable roles in regulating the complex tumor microenvironment [10–12].

The vast majority of patients diagnosed with NPC is already in the middle-late stage due to pathogenic sites is hidden and lack of effective early diagnosis methods. To find blood biomarkers for noninvasive early diagnosis of NPC, we determined 19 plasma cytokines and chemokines by luminex technology in 39 NPC patients, which contained 10 patients with stage I and stage II (2 with stage I, 8 with stage II) as well as 29 age-matched advanced stages patients (12 with stage III, 17 with stage IV). NPC patients with early stage are usually younger than late stage patients in our hospital, so a relatively younger cohort than those in the global population was recruited in this study.

Our results show that 10 cytokines and chemokines increase in NPC patients, and 7 of them decrease after therapy, which might be important in the development of NPC. MPC-1 is a protein secreted by the infiltrated inflammatory cells and associated with the maturation and attraction of tumor-associated macrophages (TAMs). It have been confirmed that MPC-1 is secreted steadily and continuously in mouse mammary tumor, and over expression of MPC-1 can directly inhibit T cells to secret IFN-γ [13, 14]. Besides, macrophage infiltration has be stunted after blocking the binding of MCP-1 to monocytes expressing the receptor of CCL2 (CCR2), which let mice to delay the progression of metastatic cancer and extend survival [15]. In our study, the expression level of MPC-1 increase in NPC patients and decrease after treatment significantly. More interestingly, we firstly find that the expression level of MPC-1 is significantly associated with the clinical stage of NPC. This result is consistent with a previous study, which has proved that MCP-1 play a role in improving the proliferation, migration and invasion of prostate cancer cells [16]. Consequently, the results suggest MCP-1 play a very important role in the growth, invasion and metastasis of NPC.

NPC is known as a highly malignant and frequently metastasized tumor with a poor prognosis [17]. The regulatory mechanism of cytokines and chemokines effecting on tumor growth, invasion, metastasis and deterioration is complicated and remains unknown. The aim of this study is to explore special cytokines and chemokines which may make a contribution to providing new strategies for the diagnosis and treatment of NPC as well as novel insights in anti-NPC immunity.

## Conclusion

Nineteen Plasma cytokines/chemokines in NPC patients before and after therapy were detected by luminex technology. The expression levels of 10 cytokines/chemokines increase in NPC patients, and 7 of them decrease after treatment with IMRT, which might be important biomarkers in the development of NPC. More interestingly, the expression level of MPC-1 is significantly associated with clinical stage suggest that MCP-1 might play a very important role in the growth, invasion and metastasis of NPC.
